# Metabolic syndrome and its association with physical activity in Sudanese early adolescents – Khartoum state, Sudan: An observational study

**DOI:** 10.1097/MD.0000000000038242

**Published:** 2024-06-07

**Authors:** Fatima A. Elfaki, Aziza I. G. Mukhayer, Mohamed E. Moukhyer, Rama M. Chandika, Husameldin E. Khalafalla, Stef P. J. Kremers

**Affiliations:** a Department of Clinical Nutrition, Applied Medical Sciences College, Jazan University, Jazan, Saudi Arabia; b Nutrim, Research Institute of Nutrition and Translational Research in Metabolism, Maastricht University, Maastricht, The Netherlands; c Department of Health Education and Promotion, Maastricht University, Maastricht, The Netherlands; d Department of Human Nutrition, School of Medicine, Ahfad University for Women, Omdurman, Sudan; e Department of Emergency Medical Services, Applied Medical Sciences College, Jazan University, Jazan, Saudi Arabia; f Department of Public Health, School of Medicine, University of Limerick, Limerick, Ireland.

**Keywords:** Global Physical Activity Questionnaire, metabolic equivalents, metabolic syndrome, physical activity

## Abstract

The growing prevalence of overweight/obesity in adolescents highlights the significance of studying metabolic syndrome (MetS) in increasingly sedentary adolescents. To date, no study in Sudan has examined the association between MetS and physical activity (PA) among adolescents. This study aimed to assess the association between MetS and its components and PA among Sudanese early adolescents. A cross-sectional assessment was conducted from to 2018 to 2019 on a sample of 921 primary school students from Khartoum State, Republic of Sudan. MetS was defined according to the International Diabetes Federation criteria and a standardized questionnaire was used to assess PA. Metabolic equivalents of task were calculated, and levels of different intensities of PA (tertiles) were identified accordingly. The association between PA and MetS and its components was assessed using a logistic regression model. The participants comprised 388 boys and 533 girls with a mean age of 12.59 ± 1.21 years. The prevalence of MetS was significantly higher in subjects in the 1st PA tertile (least active) than in the 2nd and 3rd (most active) tertiles of PA, and this difference was observed in both boys and girls. After adjusting for other study factors, the odds of MetS among adolescents in the 1st PA tertile were 7 times higher than those in the highest PA tertile (adjusted odds ratio = 7.01, 95% confidence interval: 1.48, 32.99). A physically inactive lifestyle was associated with higher odds of MetS and its components, especially waist circumference and triglyceride levels, in Sudanese early adolescents. This study highlights the importance of promoting PA in this age group.

## 1. Introduction

Globally, children and adolescents are at risk of metabolic syndrome (MetS), which is characterized by a cluster of metabolic abnormalities. It comprises a collection of risk factors such as abdominal obesity, insulin resistance, dyslipidemia, and high blood pressure (BP). These factors have long-term health implications, increasing the likelihood of cardiovascular diseases and type 2 diabetes later in life.^[1,2]^ In developing countries, the prevalence of obesity and MetS has increased rapidly, leading to increased morbidity and mortality from cardiovascular diseases and type 2 diabetes mellitus.^[3]^

Like other developing countries, Sudan has recently experienced a rapid socio-cultural change accompanied by significant shifts in lifestyle patterns.^[4]^ Due to urbanization, the availability of opportunities for regular physical activity (PA) has decreased, and leisure time activities for children and adolescents have shifted toward more sedentary pursuits, such as watching television (TV), playing computer games, and using social media.^[5]^ Concurrently, inadequate sleep, influenced by increased screen time and reduced PA, has been linked to MetS components including obesity, insulin resistance, and hypertension. All of these behavioral, cultural, and dietary changes may be the reason for excessive energy consumption by children and adolescents, weight gain, and the subsequent increase in the prevalence of obesity-related diseases, including MetS.^[2,6–9]^

PA is defined as any bodily movement produced by the skeletal muscles that requires energy expenditure. It refers to all movements, including during leisure time, for transport to and from places, or as part of a person’s work.^[10]^ Both moderate-intensity and vigorous-intensity PA improve health. Popular ways to be active include walking, cycling, wheeling, sports, active recreation, and play, and can be performed at any level of skill and enjoyment by everybody. PA is a fundamental aspect of metabolic health that provides a wide range of physiological and metabolic benefits.

Physical inactivity is a significant contributor to MetS development, leading to weight gain and the exacerbation of insulin resistance. Engaging in regular PA is essential for the management and prevention of MetS. Exercise helps control weight, improves insulin sensitivity, and regulates BP and lipid levels, positively impacting metabolic processes and reducing inflammation.^[11]^

Numerous studies have consistently associated regular PA with a reduced risk of various metabolic conditions, including MetS, type 2 diabetes, and cardiovascular diseases.^[12]^ Notably, exercise plays a crucial role in reducing the cardiometabolic risk associated with obesity, as observed in several observational studies. Both cardiorespiratory and muscular fitness (CRF) have been shown to protect against the metabolic risks associated with obesity.^[13]^

For children and adolescents, PA is essential not only for normal growth and development but also for preventing overweight and obesity and mitigating associated health risks.^[14,15]^ PA significantly reduces the risk of obesity-related health issues, such as type 2 diabetes mellitus, cardiovascular diseases, and bone health problems, as well as all-cause mortality.^[16]^ Furthermore, maintaining a healthy weight status is crucial for overall health, and PA plays a pivotal role in achieving this goal by influencing various health outcomes, including a reduced risk of cardiometabolic and vascular diseases.^[14,15,17]^

Research suggests that excessive TV and computer time can significantly increase the risk of MetS.^[18]^ According to Dunstan et al^[19]^ findings, every additional hour of daily TV viewing led to a 26% increase in MetS prevalence among women. This negative impact on health due to sedentary behavior was nearly equivalent to the positive effect of adding 30 minutes of PA to one’s daily routine. These estimations underscore the importance of reducing sedentary screen time and incorporating regular PA to promote overall health and mitigate the risk of MetS.^[19]^ Among children and adolescents, an average of 60 minutes/day of moderate-to-vigorous-intensity aerobic PA across the week provides health benefits.^[20]^ Promoting PA and exercise among all children and adolescents, irrespective of their weight status, is crucial in preventing excessive weight gain over time and reducing the potential health risks associated with a sedentary lifestyle.^[14,18]^ This study aimed to assess the associations between PA and MetS and its components among Sudanese early adolescents.

## 2. Methods

### 2.1. Study overview and participants

This school-based cross-sectional study, conducted from 2018 to 2019, focused on early adolescents aged 10 to 15 years living in Khartoum State, Republic of Sudan. Two areas, urban and rural, were selected using simple random sampling. Students were selected using multistage random clusters and stratified sampling methods. In total, 21 primary schools – 12 from urban areas and 9 from rural areas – were selected through cluster sampling with random number tables. Stratification was performed based on location (urban/rural) and the socioeconomic characteristics of the area, considering the proportion of different types of schools to avoid socioeconomic bias. After data cleaning, a total of 921 early adolescents with complete and accurate information (388 boys and 533 girls) aged 10 to 15 years.

### 2.2. Data collection

The questionnaire was designed specifically for this study and contained 4 domains: sociodemographic characteristics, PA information, anthropometric measurements, and biochemical measurements. The sociodemographic variables were gender, age, and PA questions adapted from the Global Physical Activity Questionnaire (GPAQ) developed by the World Health Organization (WHO).^[21]^ Anthropometrics (weight, height, and waist circumference (WC)), BP, and biochemical variables (fasting blood glucose [FBG], triglycerides [TG], and high-density lipoprotein cholesterol [HDL-C]) were collected. The questionnaire, prepared in English, was later translated into Arabic. Trained interviewers, following a workshop by qualified trainers, collected data. Reliability was tested using Cronbach alpha. Data collection, cleaning, and coding were performed according to the guidelines provided by the GPAQ.^[22]^ This study was approved by the Research Ethics Committee of the Federal Ministry of Health (Ref. No. 1-12-17). Informed consent was obtained from the parents and oral consent was obtained from all participating students.

### 2.3. Physical measurements

The comprehensive assessments of the participants included anthropometric and BP measurements. Weight was measured using a digital scale (SECA), with participants wearing lightweight clothing. Height was measured using a portable stadiometer while the participants stood barefoot. To evaluate overweight and obesity prevalence, age-specific and sex-specific body mass index (BMI) cutoff points recommended by the WHO (2020) and de Onis^[23]^ were applied. Overweight was defined as >+1SD, obesity as >+2SD, and underweight as <–2SD based on BMI-for-age and height-for-age (*z* score).

WC was measured in centimeters using unstretched tape, pinpointing the midpoint between the rib cage bottom and the area above the iliac crest, and recorded to the nearest 0.1 cm. Abdominal obesity was identified in children with a WC ≥ 90 cm percentile.

BP was measured using an automatic digital BP monitor with an adjustable arm cuff for different arm sizes. Participants, seated with their right arm resting at heart level, had their BP recorded twice within a 5-minute interval. The average systolic and diastolic values were considered, with cutoff points for systolic or diastolic BP ≥ 130 or ≥ 85, respectively.

### 2.4. Biochemical measurements

A standardized procedure was followed for blood sample collection from participants after an overnight fast of at least 8 hours. Using lithium heparin tubes for lipid profiling and fluoride oxalate tubes for FBG, a laboratory technician collected 5 mL of venous blood. Subsequently, the collected samples were centrifuged in an L500 Tabletop Low-Speed Centrifuge at 3000 rpm for 10 minutes to allow the blood to coagulate.

Quality checks were performed to ensure the accuracy of the analytical testing. Serum samples were processed using an A25 Analyzer with an enzymatic method to determine triglyceride (TG) and HDL-C levels. The A25 Analyzer, equipped with automated technology, assessed glycemia using the enzymatic glucose oxidase method to determine FBG levels. For comprehensive analysis, an A25 Biosystems SA Costa Brava 30 analyzer (Barcelona, Spain) was used. FBG, HDL-C, and TG were evaluated using standard laboratory reagents, enzymatic methods, and calorimetric methods. Serum triglyceride, fasting blood sugar, and HDL-C levels were measured using conventional enzymatic kits.

### 2.5. Definition of MetS and its components

The criteria for defining MetS in this study were based on the guidelines provided by the International Diabetes Federation (IDF). MetS was identified among children and adolescents exhibiting abdominal obesity (WC ≥ 90th percentile), along with the presence of 2 or more other clinical features: high BP (systolic BP ≥ 130 mm Hg or diastolic BP ≥ 85 mm Hg), low HDL-C (<40 mg/dL), elevated TG (≥150 mg/dL), and elevated FBG (≥100 mg/dL).^[24]^

### 2.6. PA measurements

This questionnaire collects the PA information on 3 domains (school, transportation, and recreational) and the frequency (number of days per week where PA lasted at least 10 minutes per session). Each domain is further classified into 3 subcategories (low, moderate, and vigorous). One MET is defined as the amount of oxygen consumed while sitting at rest, equivalent to 3.5 mL of oxygen per kilogram of body weight per minute. Youth compendium of physical activities was used to quantify the self-reports on PA into metabolic equivalents (MEF) of task.^[25]^ MET-time, representing the average weekly level of PA, was computed by multiplying MET values with the time spent at each activity level. Further categorized into tertiles separately for boys and girls and scored from 1 to 3 based on the 1st to the 3rd tertiles.

### 2.7. Statistical analysis

Following data cleaning, continuous variables were presented as mean ± standard deviation (SD). Student *t* test and analysis of variance (ANOVA) were applied to assess the differences between boys and girls based on tertiles. Odds ratios (OR) and their corresponding 95% confidence intervals (CI) were calculated using logistic regression models to evaluate the association between tertiles of PA (independent variable) and the presence of MetS (dependent variable). Model A: Logistic regression analysis without covariate adjustment. Model B: age-adjusted logistic regression analysis. Model C: age-adjusted and BMI-adjusted logistic regression analysis.

The variables were standardized using *z* scores to ensure comparability. All reported *P* values were 2-sided, and statistical significance was set at α = 0.05. Analyses were conducted using R software (version 4.3.1) for Windows, and the ggplot2 package was used to construct the ellipse chart.

## 3. Results

### 3.1. General background characteristics of the study participants

A total of 921 early adolescents participated in the study, comprising 388 (42.13%) boys and 533 (57.87%) girls, with a mean age of 12.59 (1.21) years, showing a significant difference between boys and girls. The mean (SD) weekly time spent on PA was slightly higher in girls 1756.54 (914.796) than boys 1750.12 (879.77) but statistically not significant. Notably, girls exhibited higher mean levels of BMI (*P* < .01) and TG (*P* < .01), while boys demonstrated higher mean levels of both systolic and diastolic BP (*P* < .01) (Table 1).

**Table 1:** Baseline characteristics of early adolescents based on gender.

Characteristics	Gender		Total
	Boys	Girls	
	Mean ± SD	Mean ± SD	Mean ± SD
Number	388	533	921
Age	12.69 ± 1.17	12.52 ± 1.24	12.59 ± 1.21*
BMI	18.03 ± 4.45	18.91 ± 4.72	18.54 ± 4.64**
PA (MET)	1750.12 ± 879.77	1756.54 ± 914.796	1753.84 ± 899.728
PA (*z* score)	−0.00 ± 0.98	0.00 ± 1.02	0.00 ± 1.0
WC	68.63 ± 11.11	69.49 ± 10.86	69.12 ± 10.97
SBP	113.14 ± 10.26	108.97 ± 11.41	110.72 ± 11.13**
DBP	76.45 ± 10.36	73.32 ± 11.18	74.64 ± 10.95**
FBG	103.04 ± 12.02	102.47 ± 11.02	102.71 ± 11.45
TG	90.94 ± 32.25	101.63 ± 37.31	97.12 ± 35.64**
HDL-C	52.36 ± 12.38	51.76 ± 12.57	52.01 ± 12.49

BMI = body mass index (kg/m^2^), DBP = diastolic blood pressure (mm Hg), FBG = fasting blood glucose (mg/dL), HDL-C = high-density cholesterol (mg/dL), PA = physical activity (MET-min/week), SBP = systolic blood pressure (mm Hg), TG = triglyceride (mg/dL), WC = waist circumference (cm).

* *P* < .05 significant.

** *P* < .01 highly significant.

### 3.2. PA tertiles by gender

For boys, the 1st tertile of PA encompassed participants engaging in <1632.30 MET minutes/week of PA, while those exceeding >2639.63 MET minutes/week were categorized in the 3rd tertile; subjects falling between these limits were considered in the 2nd tertile of PA. In girls, the corresponding limits were 1607.38 and 2706.75 MET minutes/week, respectively.

Table 2 presents the components of MetS according to the tertiles of PA and shows that in both sexes, WC and serum TG levels were significantly higher in subjects with the lowest PA than in the other 2 groups. Altogether 21 (2.3%) prevalence was observed among 921 early adolescents. More than two-thirds 15 (71.4%) of MetS children were in the 1st tertile of PA. Moreover, in the overall population, and in both sexes individually, the prevalence of MetS was significantly higher in subjects within the 1st tertile compared to those in the 2nd and 3rd tertiles of PA.

**Table 2:** Participants characteristics according to the physical activity tertiles by gender.

Variables	Boys tertiles			Girls tertiles			All tertiles		
	1st	2nd	3rd	1st	2nd	3rd	1st	2nd	3rd
	Mean (SD)	Mean (SD)	Mean (SD)	Mean (SD)	Mean (SD)	Mean (SD)	Mean (SD)	Mean (SD)	Mean (SD)
Number	129	122	137	173	185	175	302	307	312
Age	12.91 (1.02)	12.56 (1.24)	12.58 (1.19)*	12.44 (1.28)	12.54 (1.21)	12.58 (1.23)	12.64 (1.20)	12.54 (1.22)	12.58 (1.21)
BMI	19.02 (5.23)	17.74 (4.49)	17.36 (3.46)**	18.79 (4.81)	18.65 (4.62)	19.29 (4.75)	18.89 (5.01)	18.29 (4.59)	18.44 (4.33)
MET-PA	916.88 (262.40)	1632.30 (186.33)	2639.63 (799.20)**	944.85 (242.71)	1607.38 (184.35)	2706.75 (945.36)**	938.63 (251.585)	1617.28 (185.23)	2677.28 (883.44)**
WC	71.24 (12.24)	68.25 (11.01)	66.51 (9.55)**	70.02 (10.91)	69.73 (10.48)	68.70 (11.22)	70.54 (11.50)	69.14 (10.70)	67.73 (10.56)**
SBP	113.49 (10.31)	113.37 (9.87)	112.60 (10.60)	108.46 (11.80)	109.01 (11.57)	109.42 (10.89)	110.61 (11.44)	110.74 (11.12)	110.82 (10.86)
DBP	75.89 (10.18)	76.55 (9.60)	76.88 (11.19)	71.72 (11.43)	73.65 (10.73)	74.56 (11.27)	73.50 (11.09)	74.80 (10.38)	75.58 (11.28)
FBG	103.21 (12.62)	101.65 (11.20)	104.12 (12.12)	101.99 (9.97)	101.79 (11.38)	103.67 (11.56)	102.51 (11.18)	101.73 (11.29)	103.87 (11.79)
TG	95.29 (31.50)	92.66 (30.76)	85.32 (33.63)*	105.23 (39.69)	101.32 (36.14)	98.39 (35.99)	100.98 (36.69)	97.88 (34.31)	92.65 (35.52)*
HDL-C	52.80 (11.73)	51.81 (13. 03)	52.42 (12.46)	52.77 (13.06)	52.17 (12.46)	50.34 (12.13)	52.78 (12.49)	52.03 (12.67)	51.25 (12.30)
MetS (%)	9 (69.23%)*	2 (15.38)	2 (15.38)	6 (70.0%)*	2 (25.0%)	0 (0.00%)	15 (71.43%)*	4 (19.05%)	2 (9.52%)

BMI = body mass index (kg/m^2^), DBP = diastolic blood pressure (mm Hg), FBG = fasting blood glucose (mg/dL), HDL-C = high-density cholesterol (mg/dL), MetS (%) = number (percentage) of participants with metabolic syndrome in each tertile, PA = physical activity (MET min/week), SBP = systolic blood pressure (mm Hg), TG = triglyceride (mg/dL), WC = waist circumference (cm).

Data are mean ± SD or percentage.

* *P* < .01.

### 3.3. PA levels and MetS

Table 3 depicts the OR (95% CI) for the occurrence of MetS according to tertiles of PA in the 4 logistic regression models (Models A, B, C, and D). After adjusting for confounding factors, age, BMI, and sex in Model D, the odds of MetS among adolescents in the PA 1st tertiles were 7 times higher than those in the PA 3rd tertiles (AOR = 7.01, 95% CI: 1.48, 32.99). Overall, low PA levels significantly increased the likelihood of developing MetS.

**Table 3:** Logistic regression of physical activity levels and the metabolic syndrome.

Tertiles of physical activity	Metabolic syndrome
Model A	Odds ratio (95% CI)
3rd	Ref.
2nd	2.05 (0.37, 11.25)
1st	8.10 (1.84, 35.73)**
Model B	
3rd	Ref.
2nd	2.06 (0.38, 11.35)
1st	8.02 (1.82, 35.42)**
Model C	
3rd	Ref.
2nd	1.93 (0.32, 11.58)
1st	7.66 (1.61, 36.56)*
Model D	
3rd	Ref.
2nd	1.78 (0.30, 10.69)
1st	7.01 (1.48, 32.99)*

Model A: Logistic regression analysis without adjustment for covariates.

Model B: Age-adjusted logistic regression analysis.

Model C: Age-adjusted and BMI-adjusted logistic regression analysis.

Model D: Age- BMI and gender-adjusted logistic regression analysis.

* *P* < .05 significant.

** *P* < .01 highly significant.

### 3.4. PA and BMI among and MetS early adolescents

**Figure 1.**
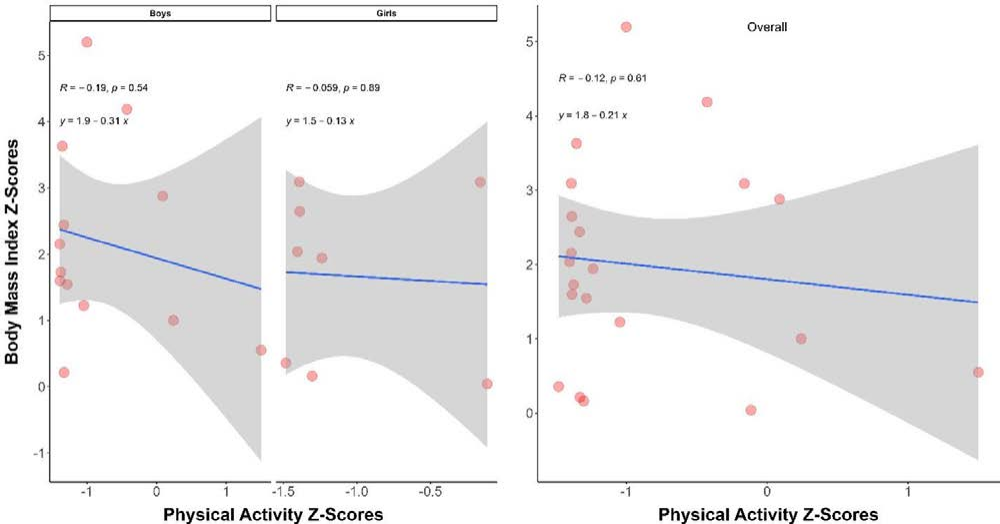
Physical activity and BMI among MetS early adolescents. Regression function of *y* = *a* + *b x*, here “*y*” = body mass index *z* scores, “*x*” = Physical activity *z* scores, “*a*” = intercept parameter and “*b*” = slope parameter, “*R*” = correlation coefficient. *Z* test for correlation coefficient conducted for testing the significance. *P* < .05 significant.

Figure 1 illustrates the plot of the predicted linear relationship between PA and BMI in early adolescents with MetS. Children’s BMI and PA values were transformed into *z* scores for visibility purposes. Although the relationship was statistically nonsignificant, the figure graphically represents the negative linear relationship between BMI and PA in both girls and boys, even among those already classified as having MetS.

## 4. Discussion

This study contributes to the existing body of research by examining the relationship between MetS and PA in Sudanese early adolescents. It is the first to study the phenomenon among Sudanese adolescents. The results of this study underscore the potential role of PA that may have a protective effect in mitigating the consequences of MetS in adolescents in Khartoum, Sudan.

The results revealed a significant association between lower PA levels and an increased likelihood of MetS among Sudanese early adolescents. This finding coincides with numerous prior studies, including those that found^[26–30]^ or PA and^[28]^ to be negatively associated with MetS. Studies focusing solely on CRF also corroborate this finding, emphasizing its negative association with MetS.^[31–35]^ A comprehensive systematic review and meta-analysis by de Oliveira and Guedes^[7]^ further supported the results, establishing a significant association between low levels of both PA and CRF and the development of MetS.

The present study observed sex-based disparities in the 3rd tertile cutoff MET minutes/week, with higher values in girls and lower values in boys. In contrast, Kelishadi et al^[36]^ found Iranian schoolboys to be significantly more physically active than girls. However, longitudinal studies are warranted to validate and elucidate these differences.

The effect of PA on the lipid profile, fat distribution, and weight status has been well explored in the literature. PA was found to reduce visceral fat^[37]^ independent of weight status.^[38,39]^ In our investigation, we observed a higher WC in the first tertile of PA than in the 3rd tertile in boys and the total population. Kang et al^[40]^ also reported a higher WC in the *inactive* group, although this disparity was not statistically significant. Nevertheless, when they compared CFR, the *unfit group* exhibited a notably higher WC than the *fit group*, and this difference reached statistical significance.^[40]^

Furthermore, our study identified higher triglyceride levels in the first tertile of PA within the total population and among boys, consistent with Kang et al findings of elevated TG levels in the physically inactive group.

The positive effect of PA on BP and/or arterial stiffness was reported in a recent systematic review.^[41–43]^ However, in our study, both systolic and diastolic blood pressure (SBP and DBP) did not exhibit a statistically significant difference between the tertiles of PA. Exercise was found to improve insulin sensitivity and reduce FBG levels.^[44–46]^ In our study, however, no difference was noted in the different tertiles of PA with respect to FBG levels. Regarding HDL-C level, our study showed no association with PA. Pereira et al^[47]^ however, reported a positive association between PA and HDL-C, and Monios et al^[48]^ found an inverse association between screen time and HDL-C.

Contrary to the existing literature, our study did not find a statistically significant difference in both systolic and diastolic BP between tertiles of PA. Previous research has consistently reported positive effects of PA on BP and arterial stiffness. However, our findings differ, highlighting the nuanced relationship between PA and BP among early Sudanese adolescents. Further exploration of these findings might help better understand BP values and variability amongst the Sudanese adolescent population.

In our study, no significant differences were observed in FBG levels across tertiles of PA. Although exercise has been recognized for its positive impact on insulin sensitivity and reduced FBG levels, our findings suggest a nuanced relationship in this population. Surprisingly, our study did not establish an association between PA and HDL-C, contrary to the findings of Pereira et al^[47]^ who reported a positive association. Moreover, Monios et al^[48]^ found an inverse association between screen time and HDL-C levels. The varied results observed in previous studies may be due to the different populations with diverse features, such as underlying health issues, genetic variables, and lifestyle choices. These differences may influence the association between PA and HDL-C level.

## 5. Limitation

Considering the inherent limitations of a cross-sectional design, caution is warranted when interpreting the results related to variations in MetS components based on PA levels. Future longitudinal and intervention studies with larger sample sizes are imperative to unravel the complex dynamics among Sudanese early adolescents.

## 6. Conclusions

This is a pioneering study in exploring MetS and its correlates among adolescents in Khartoum, Sudan. The study revealed a statistically significant association between MetS and lower PA levels among the study participants. Particularly among boys, TG and WC, key components of MetS, were significantly lower in relatively active adolescents. This study aligns with existing evidence highlighting the association between MetS and certain PA components.

Emphasizing the importance of promoting PA in this age group, our results underscore the potential role of an active lifestyle in mitigating the consequences of MetS in adolescents in Khartoum, Sudan.

## Acknowledgments

We are grateful to the schoolchildren and their parents who participated in this study. Furthermore, we would like to extend our appreciation to the research team, data collectors, data entry, and school administration. Special thanks to Adil Elfaki for his excellent administrative work and support.

## Author contributions

**Conceptualization:** Fatima A. Elfaki, Stef P. J. Kremers.

**Methodology:** Fatima A. Elfaki, Rama M. Chandika, Stef P. J. Kremers.

**Project administration:** Fatima A. Elfaki.

**Resources:** Fatima A. Elfaki.

**Writing – original draft:** Fatima A. Elfaki, Aziza I. G. Mukhayer, Mohamed E. Moukhyer, Rama M. Chandika, Husameldin E. Khalafalla, Stef P. J. Kremers.

**Writing – review & editing:** Fatima A. Elfaki, Aziza I. G. Mukhayer, Mohamed E. Moukhyer, Rama M. Chandika, Husameldin E. Khalafalla, Stef P. J. Kremers.

**Investigation:** Aziza I. G. Mukhayer, Rama M. Chandika, Husameldin E. Khalafalla.

**Supervision:** Aziza I. G. Mukhayer, Mohamed E. Moukhyer, Stef P. J. Kremers.
